# Insurance Coverage of Dermabrasion and Chemical Peel Procedures: A Critical Analysis of 58 American Insurance Companies

**DOI:** 10.7759/cureus.33184

**Published:** 2022-12-31

**Authors:** Michael Ha, Emily R Finkelstein, Mark Wieland, Aasheen Qadri, Madeline Brown, Jason Ejimogu, Yvonne M Rasko

**Affiliations:** 1 Department of Surgery, Division of Plastic and Reconstructive Surgery, University of Maryland School of Medicine, Baltimore, USA; 2 Department of Surgery, Division of Plastic and Reconstructive Surgery, University of Miami Miller School of Medicine, Miami, USA

**Keywords:** acne treatment, skin cancers, chemical peel, dermabrasion, insurance coverage

## Abstract

Introduction

Dermabrasion and chemical peels are infrequently utilized methods of treatment for medical-grade conditions despite demonstrations of favorable outcomes. Insurance coverage status has previously been shown to impact availability and accessibility to specific treatments. The purpose of this study is to evaluate the rate of insurance coverage provided for dermabrasion and chemical peel procedures in the treatment of acne, acne scarring, and non-melanoma skin cancers (NMSC).

Methods

A cross-sectional analysis of 58 insurance companies by web-based search or phone interview determined the number of insurers with a publicly available policy on dermabrasion or chemical peels. Coverage status and any corresponding criteria were extracted from existing company policies.

Results

Thirteen (22%) and 22 (38%) policies discussed dermabrasion in the treatment of basal cell carcinoma and actinic keratosis, with 62% and 73% of these policies providing coverage. Acne scarring was discussed in significantly more dermabrasion policies than basal cell carcinoma (45% vs 22%; p=0.018). However, significantly more insurers denied coverage of dermabrasion for active acne and acne scarring when compared to dermabrasion to treat basal cell carcinoma or actinic keratosis (p<0.001). Eighty-seven percent of companies (n=20) with a chemical peel policy for premalignant lesions would provide coverage, with required criteria present in 95% (n=19) of the policies that would cover chemical peels for actinic keratosis specifically. Of the 25 companies (43%) that discussed the treatment of acne with chemical peel procedures, 14 (56%) provided coverage, and 11 (44%) denied coverage. Coverage was denied by significantly less insurers for the treatment of active acne with chemical peel procedures compared to treatment with dermabrasion (44% vs 83%; p<0.006).

Conclusion

Significant discrepancies were noted in both the presence of a public policy and the coverage status of dermabrasion or chemical peel procedures among the United States health insurance companies. These inconsistencies, along with multiple criteria required for coverage, may create an artificial barrier to receiving care for specific medical-grade conditions.

## Introduction

Dermabrasion and chemical peel procedures are popular methods of skin resurfacing performed in the United States by dermatologists and plastic surgeons [1]. These skin resurfacing techniques improve skin quality, texture, and appearance by ablating part of the epidermis or epidermis and superficial dermis, allowing for subsequent regeneration and reepithelization [1]. The high concentration of pilosebaceous glands and rich vascular networks of the face makes it an excellent area for the wound healing processes that are necessary to achieve these desired results following ablation [2].

Skin resurfacing procedures such as dermabrasion and chemical peels can be therapeutic for nonmelanoma skin cancers (NMSC), with clearance of precancerous or cancerous lesions demonstrated in up to 96% of patients [3-10]. These procedures may also play a large role in protection against the development of new precancerous lesions [3-5,7-10]. In patients suffering from chronic acne, studies have investigated resurfacing procedures as either monotherapy or complementary treatment to current acne regimens. These prospective studies have shown statistically significant reductions in active acne lesions up to 12 weeks following skin resurfacing, suggesting a potential longevity and cost-effective aspect of this treatment [11-13]. Furthermore, dermabrasion and chemical peel procedures have demonstrated substantial reductions in the appearance of acne scarring that occurs with longstanding disease [11,14-16].

Despite being recognized forms of treatment, dermabrasion and chemical peel procedures are uncommonly selected as a treatment for acne, acne scarring, and various types of NMSC [10]. A potential barrier to receiving these procedures may be the cost, which could be discouraging patients from obtaining this form of treatment [17,18]. Previous literature has recognized that low rates of insurance coverage and variable criteria can hinder a patient’s ability to receive certain treatments due to substantial associated out-of-pocket costs [19,20]. To further investigate cost as a limiting factor to receiving dermabrasion and chemical peel procedures, we evaluated the number of insurance companies that will cover treatment with skin resurfacing, and whether the required criteria present in policies may be a barrier to receiving care. This article was previously posted to the Research Square preprint server on November 15, 2022.

## Materials and methods

This study was exempted from Institutional Review Board approval at our institution. Fifty-eight American insurance companies were selected and determined to be representative of the vast majority of Americans with health insurance. To begin the selection of these companies, we collected the top 50 insurers with the greatest market share in the United States [21,22]. This list of companies was then cross-referenced with the principal insurer per enrolment in each state, leading to an additional eight companies to undergo evaluation.

Separate web-based searches were conducted to determine whether each company had a publicly available policy on dermabrasion and chemical peel procedures. If no policy was identified by a web-based search, a phone call or email to the company was made to locate the public policy, if available. All companies with a publicly available policy were further evaluated for an insurance coverage status for specific medical indications. Any applicable Current Procedural Terminology (CPT) codes related to dermabrasion and chemical peel procedures were also extracted from policies at this time. Companies that did not have a statement regarding coverage but included corresponding covered or non-covered CPT codes were grouped with those companies that had a policy for the purposes of this study.

The coverage status of each company with a public policy was sorted into one of three categories: covered with or without criteria; not covered; or covered on a case-by-case basis. An insurer was considered to not cover treatment for a given indication when the company declared that coverage would not be provided under any circumstances. Companies were grouped into the coverage on a case-by-case basis category only if this type of coverage was explicitly stated in the policy. For companies that would provide coverage, any required criteria were extracted and categorized.

All data were compiled and analyzed using Microsoft Excel (Microsoft Corp., Redmond, WA). Either a Chi-squared test or Fisher’s exact test was used to compare categorical variables when appropriate. Statistical significance was defined as a value of p<0.050.

## Results

Fifty-eight insurance companies including Medicare and Medicaid were evaluated in this study. Forty-seven of these insurers (81%) had a publicly available policy that included at least one statement on coverage on either dermabrasion or chemical peel procedures to treat acne, acne scarring, or non-melanoma skin cancers (NMSC).

Dermabrasion

**Figure 1:**
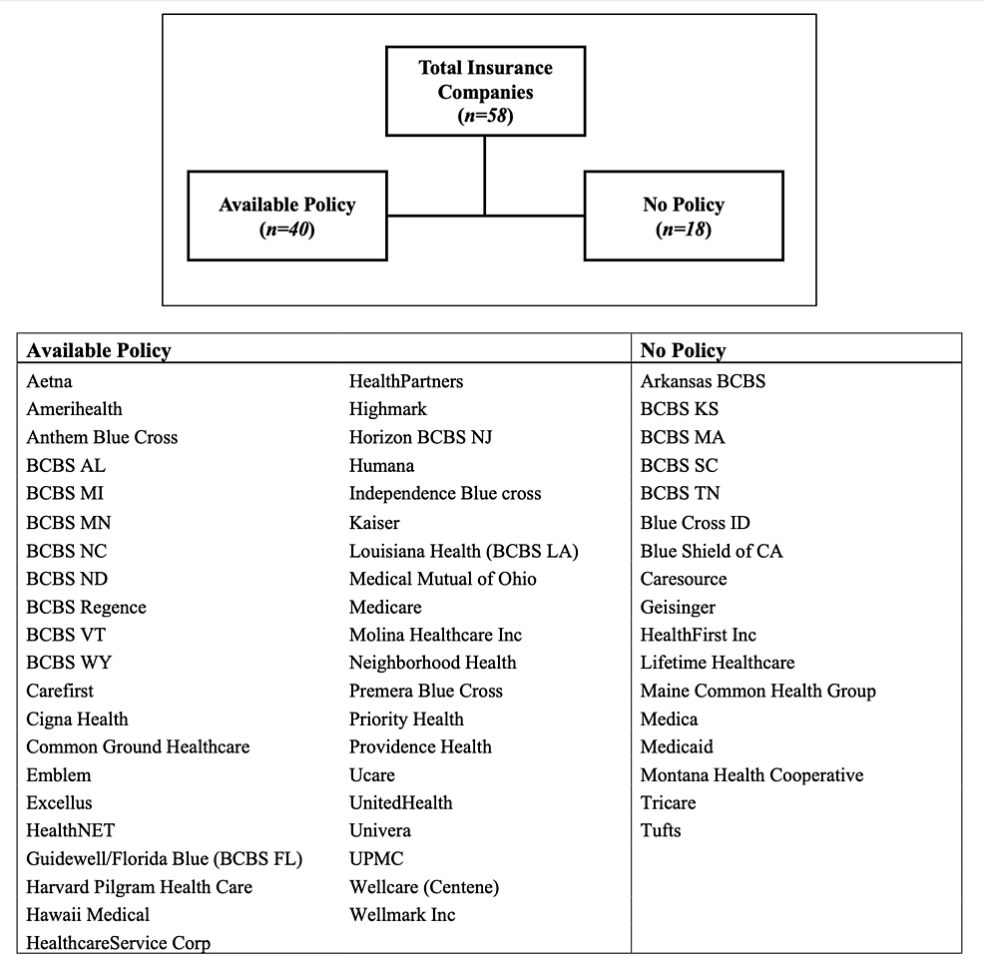
Insurance companies with a public policy on dermabrasion procedures BCBS: Blue Cross Blue Shield; UPMC: University of Pittsburgh Medical Center; Corp: Corporation; Inc: Incorporated; AL: Alabama; MI: Michigan; MN: Minnesota; NC: North Carolina; ND: North Dakota; VT: Vermont; WY: Wyoming; FL: Florida; NJ: New Jersey; LA: Louisiana; KS: Kansas; MA: Massachusetts; SC: South Carolina; TN: Tennessee; ID: Idaho; CA: California

**Figure 2:**
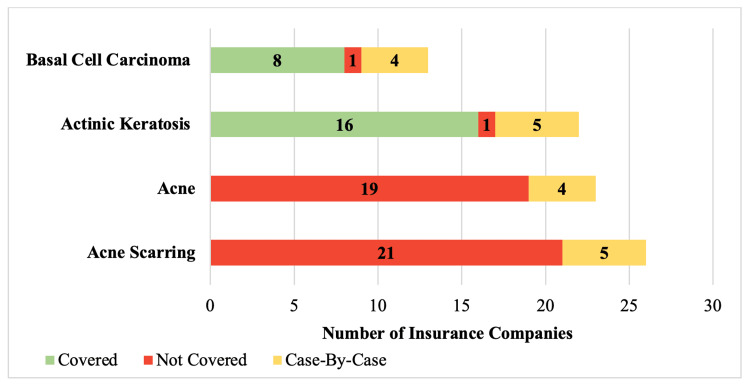
Insurance company coverage of dermabrasion for specific medical indications

Forty of the 58 insurance companies (69%) included at least one statement on coverage for dermabrasion specifically (Figure 1). Thirteen (22%) and 22 (38%) policies discussed dermabrasion for the treatment of basal cell carcinoma and actinic keratosis. There was no significant difference in the frequency of inclusion within policies (22% vs 38%; p=0.106) or the number of insurance companies offering to provide coverage for dermabrasion (62% vs 73%; p=0.755) between these indications (Figure 2). Criteria to qualify for coverage were required by five of the companies (63%) that covered basal cell carcinoma and nine of the companies (56%) that provided coverage for actinic keratosis (Table 1).

 

**Table 1:** Criteria required for coverage of dermabrasion for the management of basal cell carcinoma and actinic keratosis BCC: basal cell carcinoma; AK: actinic keratosis; 5-FU: 5-fluorouracil; No.: number

Criteria for Coverage	No. Policies	
	BCC	AK
Cryotherapy, curettage, and excision are impractical	4	6
Prior failed trial: topical retinoid, topical chemotherapeutic agents, or cryotherapy	4	8
Documented evidence of 10 or more BCC or AK	1	4

Twenty-three (40%) and 26 (45%) insurance companies incorporated a statement on coverage of dermabrasion as a treatment for acne and acne scarring. Dermabrasion for acne scarring was discussed in policies significantly more often than dermabrasion for the treatment of basal cell carcinoma (45% vs 22%; p=0.018). Coverage would be denied significantly more often for both active acne and acne scarring than it would be for either basal cell carcinoma or actinic keratosis (p<0.001) (Figure 2). Furthermore, no companies would extend coverage for dermabrasion when used as management for acne or acne scarring. 

Sixteen insurers (28%) denied coverage for dermabrasion if performed for the following indications: wrinkling of the skin (n=10), uneven pigmentation (n=10), non-traumatic tattoo removal (n=4), rosacea (n=4), photoaged skin (n=3), traumatic scar revision (n=2), and melasma (n=2). Four companies (7%) would cover these additional indications: previous trauma (Highmark), certain scar revisions (Independence Blue Cross), rhinophyma (Medicare), and restoration following a medically necessary procedure (Neighborhood Health). Thirty companies (52%) had CPT codes listed in a policy related to one of the indications under investigation in this study (Table 2). 

**Table 2:** CPT codes for dermabrasion included in company policies CPT: current procedural terminology

CPT Codes	Code Description	Covered Codes	Denied Codes
15780	Dermabrasion; total face	22	6
15781	Dermabrasion; segmental, face	23	6
15782	Dermabrasion; regional, other than face	22	6
15783	Dermabrasion; superficial, any site	19	7
15786	Surgery, integumentary - scraping of skin growth	7	1
15787	Surgery, integumentary - scraping multiple growths	6	1

Chemical peels

**Figure 3:**
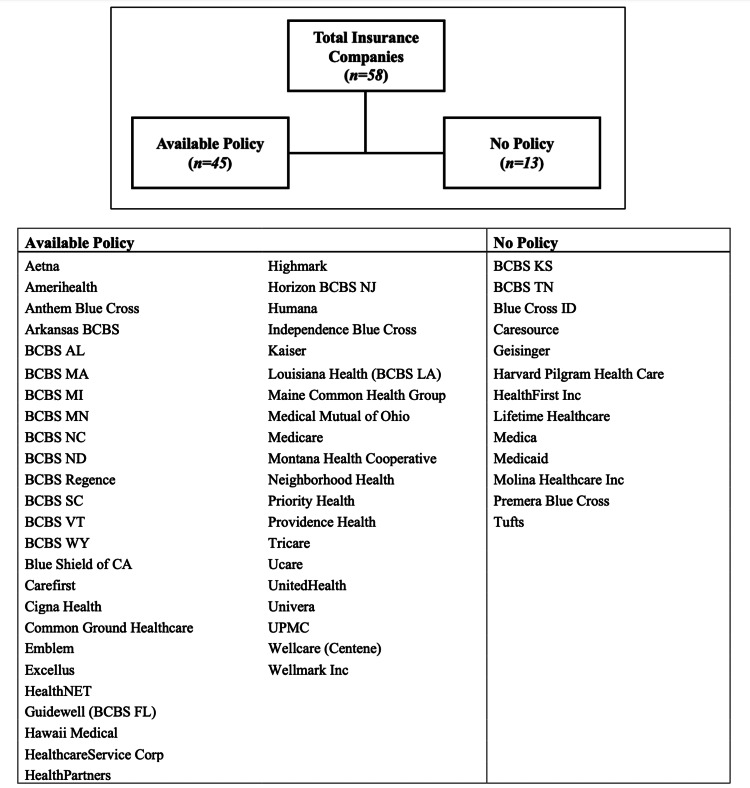
Insurance companies with a public policy on chemical peel procedures BCBS: Blue Cross Blue Shield; UPMC: University of Pittsburgh Medical Center; Corp: Corporation; Inc: Incorporated; AL: Alabama; MI: Michigan; MN: Minnesota; NC: North Carolina; ND: North Dakota; VT: Vermont; WY: Wyoming; FL: Florida; NJ: New Jersey; LA: Louisiana; KS: Kansas; MA: Massachusetts; SC: South Carolina; TN: Tennessee; ID: Idaho; CA: California

**Figure 4:**
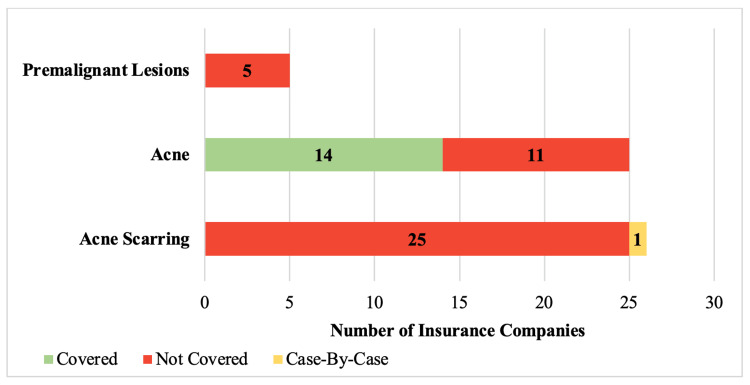
Insurance company coverage of chemical peel procedures for specific medical indications

Forty-five insurance companies (78%) incorporated a statement on coverage of chemical peels within a company policy (Figure 3). Of the 23 insurers (40%) that discussed coverage of chemical peel treatments for actinic keratosis, 20 companies (87%) would provide coverage (Figure 4). This did not significantly differ from the proportion of insurance companies providing coverage for dermabrasion if performed for this indication (87% vs 73%; p=0.412). Criteria required for coverage were present in 95% (n=19) of the policies that covered chemical peels for the management of actinic keratosis (Table 3). 

**Table 3:** Required criteria for coverage of chemical peel procedures for premalignant lesions and active acne No.: number; AK: actinic keratosis; BCC: basal cell carcinoma; 5-FU: 5-fluorouracil

Indication	Criteria for Coverage	Criteria Stratification	No. Policies
AK and BCC			
	Greater than 10 lesions		18
	Prior failed trial of other therapies		9
		Topical 5-FU or imiquimod ineffective or contraindicated	6
Active Acne			
	Prior failed trial of other therapies		13
		Topical or oral antibiotics ineffective or contraindicated	12
	Use of epidermal peels		10
		Use of 40-70% alpha hydroxy acids	5
	To treat comedomal acne		4

Chemical peel procedures to treat active acne were included in 25 (43%) insurance company policies, being covered by 14 (56%) companies, and denied coverage in the remaining 11 (44%). Significantly fewer companies would deny coverage of chemical peels for the treatment of active acne when compared to dermabrasion (44% vs 83%; p<0.006). Of the companies that provided coverage for this indication, all but one company (n=13, 93%) had required criteria to be met before coverage would be granted (Table 3). Twenty-six insurance companies (45%) discussed coverage of chemical peels for the treatment of acne scarring, with significantly more companies denying coverage for this indication when compared to active acne (n=25 vs n=11; p<0.001). No insurers would extend coverage for chemical peels when used as management for acne scarring. 

Nineteen companies (33%) denied coverage for dermabrasion if performed for the following indications: wrinkling (n=17), photoaged skin (n=15), uneven pigmentation (n=7), lentigines (n=3), and rosacea (n=2). Two companies (Highmark & Independence Blue Cross) provided alternative indications that would be considered for reimbursement, being rosacea and irregularities caused by trauma or accidents. Thirty-three companies (57%) had CPT codes listed in a policy related to one of the indications under investigation in this study (Table 4).

**Table 4:** CPT codes for chemical peel procedures included in company policies CPT: current procedural terminology

CPT Codes	Code Description	Covered Codes	Denied Codes
15788	Chemical peel, facial; epidermis	22	9
15789	Chemical peel, facial; dermis	24	7
15792	Chemical peel, nonfacial; epidermal	21	10
15793	Chemical peel, nonfacial; dermal	24	8
17360	Chemical Exfoliation for acne	8	5

## Discussion

Most of the American insurance companies evaluated in this study provided a publicly available policy on dermabrasion or chemical peel procedures. Coverage of either procedure for the treatment of NMSC was discussed by fewer than half of the evaluated insurers, though coverage was usually provided. While no companies would cover the treatment of active acne with dermabrasion, insurers were about evenly divided on whether they would provide or deny coverage for the treatment of acne with chemical peels. In general, the majority of companies that extended coverage for active acne or NMSC have one or more criteria to be met before coverage would be provided. Our study highlights great inconsistencies in the rates of inclusion in policies and coverage of skin resurfacing procedures for medical-grade conditions between United States insurance companies. These incongruencies, along with multiple criteria required for coverage, may discourage patients from utilizing skin resurfacing procedures as a method of treatment. 

Skin cancer is the most common malignancy in the United States, with basal cell carcinoma, squamous cell carcinoma, and actinic keratoses comprising the majority of cases [23]. Over one-half of American insurers did not have a statement on whether they would provide coverage of skin resurfacing procedures for the management of NMSC, leaving the coverage status ambiguous. Surgical excision is the current mainstay of treatment for NMSC [24]. Although it has some of the lowest recurrence rates recorded in the literature, patients are at risk for undesirable cosmetic outcomes, disfigurement, and dysfunction in the operative area [5,24]. As with other well-known treatment options such as cryosurgery and 5-Fluorouracil (5-FU), skin resurfacing procedures are an alternative to surgical treatment with promising evidence of efficacy demonstrated in the literature [3-7]. In addition to infrequent treatment requirements and a low overall cost of treatment, dermabrasion and chemical peels are set apart from the other nonsurgical methods by their ability to provide both eradication and prophylaxis against future lesions [4,7,24]. Lawrence et al. demonstrated that a combination of Jessner’s solution with 35% trichloroacetic acid (TCA) is as effective as a three-week course of topical 5% 5-FU in the treatment of actinic keratosis [9]. In addition to finding that 96% of patients would remain free of lesions for one year, Coleman et al. concluded that dermabrasion may be more effective in preventing the development of new lesions than treatment with either cryosurgery or 5-FU [8]. However, when evaluating the clinical utility of skin resurfacing procedures, it is imperative to address the paucity of large randomized controlled trials conducted for the treatment of NMSC specifically [6]. Insurers may be less willing to discuss coverage of these resurfacing techniques for this reason.

Over 50 million Americans suffer from active acne, with up to 95% of these individuals experiencing some degree of residual scarring [16,23]. While no companies would cover dermabrasion for the treatment of active acne, insurers were almost evenly divided on whether coverage would be extended or denied if chemical peels were instead used for management. The literature surrounding dermabrasion tends to focus on its utility in acne scarring rather than active acne, likely contributing to the absence of insurers willing to provide coverage for the latter indication [14-16]. Alternatively, chemical peel procedures have demonstrated promising results for both the resolution and prevention of recurrent acne [11-13]. In a prospective clinical trial testing a single treatment of TCA 35%, most patients saw a statistically significant reduction in their acne by at least 75% [11]. There were no relapses of active lesions from any of the study participants at 12 weeks, demonstrating its long-term viability and possible cost-effectiveness [11]. There may also be an additive effect when multiple peels are used simultaneously, creating a higher and earlier therapeutic response that lasts longer than a single peel alone [25]. These benefits, coupled with evidence from clinical trials, may be one reason why some insurers were willing to provide coverage of chemical peels for this indication. However, several insurers still denied coverage of chemical peel treatments for active acne, considering this indication to be cosmetic. What these companies might fail to consider is the detrimental psychological distress that patients with longstanding acne frequently face. The severity of the anxiety and depression attributed to longstanding acne has been compared to that of life-threatening or physically disabling diseases [26]. With increased awareness surrounding the topic of appropriate management for mental health in recent years [27], we may see a rise in the number of insurance companies that consider the treatment of acne to be medically necessary for this reason. 

Regardless of the indication, most of the insurance companies that extended coverage for dermabrasion or chemical peels had certain criteria to fulfill before patients would be eligible for reimbursement. Among the most frequently mentioned in policies was the prerequisite of ten or more precancerous or cancerous lesions before coverage of treatment would be considered, required by 90% of policies offering coverage of chemical peel procedures. In addition to an absence of concrete guidelines for the treatment of NMSC with either skin resurfacing procedure, no insurer provided evidence from the literature supporting the requirement of ten or more lesions, leaving one to question the basis of this criterion [24]. Despite the number of lesions not being found to influence the efficacy of treatment, insurance companies may have based this requirement on the high average number of lesions that are present in certain study populations [5,9,10]. Nonetheless, there are patients in these studies with less than ten lesions that had significant reductions or eradication of their cancer following these interventions [5,9,10]. Even if a patient meets this necessary number of lesions to qualify for coverage, most available policies also required a prior failed trial of other common therapies such as topical retinoids, topical chemotherapy, or cryotherapy. Therefore, one’s ability to receive coverage for skin resurfacing procedures may also be dependent on whether the insurance company will cover the initial alternative treatments required by these trials. Another common criterion that appeared in policies was a prior failed trial of topical and oral antibiotics, required by 93% of companies that would extend coverage for chemical peels in patients with active acne. However, chemical peel procedures may be a suitable alternative for the numerous patients that struggle with acne medication noncompliance. Reasons for this noncompliance include high costs of medication, hassles associated with taking daily pills, and concerns over potential medication side effects [28,29]. While the American healthcare system spends over $1.74 billion annually on prescription drugs for acne, the average cost of a single dermabrasion and chemical peel procedure in 2020 was $1786 and $519 [17,18,30]. Infrequent treatment requirements and low overall cost in comparison make these procedures an attractive, cost-effective option for both the patient and the healthcare system at large [17,18]. In replacing the need for medication regimens, treatment with skin resurfacing procedures may benefit patients that struggle with the management of daily prescriptions. As more randomized controlled trials are performed demonstrating more definitive evidence of efficacy, we may see a shift in coverage and required criteria to reflect these advantages. 

The main limitation of this study is the cross-sectional nature of its design. We are therefore unable to account for policies that have evolved or have made periodic changes since the time of original data collection. There is also the possibility of written policy not reflecting the true coverage practices of a company. Since we were only able to account for publicly available policies and not those policies that are private, the true estimated coverage of these resurfacing treatments may be underestimated. Lastly, we did not incorporate every insurance company in the United States within our analysis. Nonetheless, this study shows overall strength due to the large number, popularity, and market share of insurance companies that were included, which together represent the majority of Americans with health insurance. 

## Conclusions

Most American insurance companies have a publicly available policy on either dermabrasion or chemical peel procedures for the treatment of acne, acne scarring, or NMSC. Although NMSC was less frequently mentioned in policies, it was usually a covered indication. On the other hand, insurers were almost equally divided on whether they would extend or deny coverage for chemical peels as a treatment for active acne. The vast majority of companies that provided coverage for this indication had one or more criteria to be met before coverage would be provided. Inconsistencies in both inclusion and coverage between insurance companies, along with various required criteria, may create an artificial barrier to receiving care.
